# Transurethral Resection of Bladder Tumors Using Holmium Laser: An Observational Study

**DOI:** 10.7759/cureus.91215

**Published:** 2025-08-28

**Authors:** Thota Siddhartha, Vasudevan T, Suvit Jumde

**Affiliations:** 1 Urology, Mahatma Gandhi Medical College and Research Institute, Puducherry, IND

**Keywords:** bladder cancer, holmium laser, minimally invasive surgery, non-muscle invasive bladder cancer, transurethral resection

## Abstract

Background

Bladder cancer ranks as the ninth most frequently diagnosed malignancy globally. Although conventional treatment, i.e., transurethral resection of the bladder tumor (TURBT), remains the gold-standard procedure for early-stage bladder cancer, it carries well-documented risks such as bleeding, perforation, and prolonged catheter dependence. In contrast, holmium laser resection promises several theoretical benefits, namely, lower thermal damage to surrounding tissues, reduced incidence of the obturator nerve reflex, and superior control of intraoperative bleeding.

Methodology

This cross-sectional observational study was conducted at the Department of Urology, Mahatma Gandhi Medical College and Research Institute, Puducherry, between December 2021 and June 2023. In total, 24 patients older than 18 years and diagnosed with superficial bladder carcinoma (Tis, Ta, T1, N0, M0) underwent holmium laser-assisted transurethral resection. Operative time, complication rates, duration of catheterization, length of hospital stay, and histological results were systematically recorded and analyzed.

Results

The study population comprised 62.5% males and 37.5% females. Hematuria was the most common presenting symptom (58.3%). The majority of tumors were located in the right lateral wall (37.5%) and posterior wall (25.0%). Single lesions predominated (70.8%). The median operative time was 32.50 minutes, with a median catheterization time of two days and a hospital stay of 1.50 days. Complications included bladder perforation (8.3%) and obturator reflex (12.5%). Tumor staging showed 62.5% T1 and 37.5% Ta stages, with 58.3% classified as low-grade tumors.

Conclusions

Holmium laser-assisted TURBT demonstrated acceptable operative outcomes with relatively low complication rates, shorter hospital stays, and effective tumor resection. The technique appears to be a viable alternative to conventional TURBT for superficial bladder carcinoma management.

## Introduction

Bladder cancer is a major global malignancy, with approximately 437,000 new diagnoses and 186,000 deaths in 2016 [1]. Its incidence shows geographic variation, higher in Western Europe and North America, influenced by genetic susceptibility, occupational hazards, and environmental factors beyond smoking [2-4]. It predominantly affects older adults, with a median age at diagnosis of 69 years for men and 71 years for women, and incidence rises sharply with age. Cigarette smoking remains the primary risk factor, with relative risks of 4.0 in men and 4.7 in women compared to never-smokers [5-8]. In younger patients, tumors are often low-grade and non-invasive [9].

For non-muscle-invasive bladder cancer, standard treatment involves transurethral resection of the bladder tumor (TURBT) followed by intravesical chemotherapy or immunotherapy [10]. However, conventional monopolar TURBT (CM-TURBT) is associated with significant risks, including bleeding, obturator nerve reflex (ONR), and bladder wall perforation, particularly for tumors on the lateral wall [11]. ONR results from electrical current stimulating the obturator nerve during resection of lateral lesions, causing sudden adductor muscle contractions that may lead to deeper tissue injury or perforation. Moreover, diathermy in CM-TURBT generates high temperatures (100°C-300°C), producing a broad zone of thermal necrosis beyond the resection site [12]. In contrast, laser-based approaches offer potential advantages by eliminating electrical current, thus preventing ONR in lateral tumor resections, and operating at lower peak temperatures (40°C-75°C) with limited thermal penetration, reducing damage to surrounding muscle and nerve tissues [13]. The application of lasers in urology dates back to 1966, when Parsons et al. used a pulsed ruby laser for bladder tissue ablation, paving the way for endoscopic laser delivery via flexible quartz fibers and argon ion lasers [14,15].

Among laser technologies, the holmium:YAG (Ho:YAG) laser has gained prominence for bladder tumor resection due to its 2,100 nm wavelength, which exhibits strong water absorption, resulting in shallow tissue penetration (approximately 400 μm) and superior hemostatic effects. The laser creates steam bubbles that mechanically separate tissue layers while coagulating blood vessels, enabling precise cutting with minimal bleeding [13]. Despite these theoretical benefits and encouraging early results, clinical evidence for Ho:YAG laser in bladder cancer remains sparse, primarily from small-scale studies with heterogeneous cohorts and inconsistent outcomes [10-15]. There is a pressing need for robust observational studies to systematically evaluate its safety, efficacy, and outcomes in superficial bladder carcinoma.

This study assesses the clinical outcomes, safety profile, and technical feasibility of Ho:YAG laser-assisted TURBT in patients with superficial bladder carcinoma. Key objectives include evaluating operative parameters (e.g., resection time, hemostasis), complication rates (e.g., ONR, perforation, bleeding), recovery times, and histopathological findings to offer evidence-based insights into this innovative surgical technique.

## Materials and methods

Study design

This study followed a cross-sectional, observational framework to explore the effectiveness of holmium laser-assisted transurethral resections in individuals with superficial bladder cancer. A prospective setup enabled consistent data capture and an in-depth review of clinical variables alongside patient outcomes.

Study setting and duration

The research was conducted in the Department of Urology at Mahatma Gandhi Medical College and Research Institute (MGMCRI) in Puducherry, India. Patient recruitment and data recording took place over an 18-month period, from December 2021 to June 2023.

Study population and sample size

Men and women aged 18 or older who had superficial bladder carcinoma and were booked for TURBT in the department formed the study group. To estimate the required number of participants, the mean blood loss cited in the literature, 32.7 ± 4.6 mL, was used in combination with standard sample size equations, a 0.05 alpha level, and 90% power, yielding a target of 24 patients. Eligible individuals were then selected through a convenient, nonrandom sampling procedure.

Inclusion criteria

Eligible participants were adult patients at least 18 years old whose bladder tumors had been classified by biopsy as either carcinoma in situ, stage Ta, or stage T1 and had no evidence of distant spread. Imaging tests, such as multiplanar ultrasound, MRI, or CT, had to show that the tumors were still confined to the bladder wall, with no signs of invasion into surrounding soft tissue, lymph nodes, or nearby organs. Finally, all patients needed to be medically cleared for surgery and able to undergo the procedure under either spinal or general anesthesia.

Exclusion criteria

Patients were excluded from the study if they had muscle-invasive bladder cancer on confirmed histopathology, if they harbored non-papillary tumors larger than 3 cm in diameter, or if they presented with concurrent urological conditions such as obstructing urethral stones, symptomatic prostatic hyperplasia, or significant urethral stricture disease. Also excluded from participation were individuals with serious systemic illnesses, mainly active severe infection, end-stage renal failure, and decompensated liver cirrhosis, people who had been treated for any other cancer in the past, and those who, for any reason, could not provide informed consent.

Preoperative assessment

Every patient underwent a thorough preoperative assessment, which included an extensive medical history, a complete physical examination, and standard blood and urine tests. Imaging consisted of abdominal ultrasonography, CT of the kidney, ureter, and bladder (CT-KUB), and plain X-ray KUB. Tumors were then inspected with cystoscopy for accurate visualization, precise localization, and staging. Informed written consent was secured from each individual in the language he or she preferred.

Surgical technique

Surgery was performed under either spinal or general anesthesia, with the choice guided by the anesthesiology team and the patient’s own wishes. Holmium laser-assisted en bloc resection of bladder tumors (HoL-EBRBT) was done with a 100-W Lumenis unit and a 550-µ end-firing fiber, delivered through a 26-F resectoscope (Richard Wolf, Knittlingen, Germany).

Laser settings were fixed at 1-2 J and 40-50 Hz, and the tumor was removed as a single piece with at least a 1-cm margin. Normal saline was used for continuous irrigation throughout the procedure. After resection, multiple biopsies from the tumor bed and from the mucosal edges were taken at two different sites for histopathological review.

Surgeon experience and standardization

All procedures were performed by two experienced urologists (>5 years post-residency, >100 conventional TURBT procedures each) who completed standardized holmium laser training involving >20 supervised procedures before study initiation. A standardized operative protocol was developed jointly by both surgeons, with weekly case reviews conducted to ensure technique consistency throughout the study period.

For patients undergoing spinal anesthesia with lateral wall tumors, a prophylactic obturator nerve block was routinely performed using 0.25% bupivacaine (10 mL) under ultrasound guidance. In general anesthesia cases, adequate neuromuscular blockade was maintained using rocuronium (0.6 mg/kg) with train-of-four monitoring to prevent inadvertent muscle contractions.

Complication assessment

Bladder perforation was identified by: (1) visualization of perivesical fat during resection, (2) loss of bladder distension despite adequate irrigation flow, and (3) extravasation of irrigant into the surgical field. Suspected perforations underwent immediate cystographic confirmation when clinically indicated. ONR was defined as visible adductor muscle contraction during lateral wall resection, graded as mild (no surgical interruption) or severe (requiring procedure pause).

Postoperative management

Three-way 20-French Foley catheters were placed in all patients immediately after surgery. Normal saline bladder irrigation began at once if frank hematuria appeared and continued until the urine cleared. Mitomycin C 40 mg was given intravesically within six hours to every patient except those thought to have perforated bladders. In suspected perforation cases, patients received six weekly doses of intravesical Bacillus Calmette-Guérin (BCG) 120 mg, starting four weeks after surgery.

Routine blood tests were drawn at six and twenty-four hours to check biochemical recovery. Patients left the hospital only when hematuria had stopped and normal voiding returned after the catheter was removed. Follow-up consisted of ultrasound, urinary cytology, and cystoscopy at planned intervals to watch for complications and recurrence.

Outcome measures

The primary endpoints included total operative time, ONR events, bladder perforation, catheter days, length of hospital stay, and final histopathology. Secondary endpoints tracked analgesic consumption, length of analgesic effect, tumor pT stage, and recurrence-free survival. All clinical and pathologic staging used the published TNM 2017 classification system.

Statistical analysis

All statistical analyses were conducted in Python, with pandas handling data manipulation, NumPy supporting numerical operations, and SciPy providing the statistical tests. Continuous variables were summarized by central tendency and dispersion measures matched to their distribution. For data that met normality assumptions, mean and standard deviation were reported; median and interquartile range were used for skewed variables. Categorical data were presented as counts and percentages. To compare continuous variables across groups, the study employed the Mann-Whitney U test when distributions were non-parametric. A p-value of less than 0.05 was set as the marker for statistically significant differences. Before testing, pandas was used to clean and preprocess the data, verifying its integrity and fitness for analysis.

Ethical considerations

This observational study was conducted in the Department of Urology at MGMCRI and started only after the Institutional Ethics Committee granted approval (Registration No. MGMCRI/01/2021/2/IHEC/157). Before recruiting any participants, the research team secured broad ethical clearance, confirming that all activities met the established safety standards for patients and researchers alike. Each volunteer provided written informed consent before joining the study, a process during which the team explained the aims, procedures, and any rights the patient retained as a participant. Throughout data collection, strict measures for confidentiality and data protection were enforced to shield each participant’s private medical details from unauthorized access. All clinical procedures and research tasks followed the core principles of the Declaration of Helsinki on ethics in human research. In addition, the study met every relevant institutional guideline so that the welfare of participants remained the first priority at every stage of the investigation.

## Results

The study population consisted of 24 patients with superficial bladder carcinoma who underwent holmium laser-assisted transurethral resection. The gender distribution showed a male predominance, with 15 (62.5%) patients being male and nine (37.5%) patients being female. The presenting symptoms varied among study participants, with hematuria being the most common presentation occurring in 14 (58.3%) patients. Lower abdominal pain was reported by four (16.7%) patients, while frequent urination was observed in three (12.5%) patients. Incidental discovery of bladder tumors occurred in three (12.5%) patients.

Smoking history was documented in six (25.0%) patients, while 18 (75.0%) patients were non-smokers. The prevalence of comorbidities was significant, with diabetes mellitus being the most common comorbidity, affecting 13 patients (54.2% overall prevalence). Hypertension was present in eight (33.3%) patients, while coronary artery disease was documented in five (20.8%) patients. Only four (16.7%) patients had no documented comorbid conditions.

Tumor characteristics and distribution

The anatomical distribution of tumors showed the right lateral wall as the most common location, accounting for nine (37.5%) cases, followed by the posterior wall with six (25.0%) cases, left lateral wall with four (16.7%) cases, dome with three (12.5%) cases, and anterior wall with two (8.3%) cases.

Single lesions predominated in 17 (70.8%) patients, while seven (29.2%) patients had multiple lesions. Detailed analysis of the multiple lesions group showed four patients with two lesions, two patients with three lesions, and one patient with four lesions. Median operative time increased progressively with lesion number: two lesions (30 minutes, IQR = 28-32), three lesions (35 minutes, IQR = 33-37), and four lesions (45 minutes). The patient with four lesions required the longest catheterization (three days) and hospital stay (three days). Bladder neck involvement was documented in five (20.8%) patients, while 19 (79.2%) patients had tumors located elsewhere in the bladder. The median tumor diameter was 2.00 cm (IQR = 1.50-2.50).

Operative outcomes and complications

The median operative time was 32.50 minutes (IQR = 25.00-38.75). Analysis of the learning curve showed that the first five procedures had longer operative times (median = 45 minutes, IQR = 40-50) compared to subsequent cases (median = 30 minutes, IQR = 25-35), demonstrating successful technique adoption by both operating surgeons. ONR, a significant complication of bladder tumor resection, occurred in three (12.5%) patients, while 21 (87.5%) patients did not experience this complication. All ONR episodes were classified as mild grade (brief muscle contraction without procedural interruption) and occurred despite prophylactic obturator nerve block in spinal anesthesia cases.

Bladder perforation, another important surgical complication, was observed in two (8.3%) patients, while 22 (91.7%) patients had no perforation. Both perforations were identified intraoperatively through visualization of perivesical fat and managed conservatively without requiring surgical repair or conversion to open surgery.

Location-specific complication analysis

Among the 13 patients with lateral wall tumors (right lateral: nine, left lateral: four), ONR occurred in two (15.4%) cases despite prophylactic obturator nerve block. Both cases involved right lateral wall tumors and were managed without procedural interruption. No bladder perforations occurred in lateral wall tumor resections.

The two bladder perforations occurred in the posterior wall (n = 1, 4.2% of posterior wall cases) and dome locations (n = 1, 33.3% of dome cases). Both perforations were small (<5 mm) and managed conservatively with prolonged catheterization (five days each) and antibiotic prophylaxis. For tumors not involving lateral walls (n = 11), no ONR episodes were recorded, confirming the anatomical specificity of this complication.

Recovery parameters

The median duration of catheterization was 2.00 days (IQR = 1.00-2.00), with patients requiring perforation management having extended catheterization periods (five days each). The median length of hospital stay was 1.50 days (IQR = 1.00-2.75), and the median number of analgesic vials consumed was 1.50 (IQR = 1.00-2.00). The median duration of analgesia was 1.00 hour (IQR = 1.00-2.00).

Bladder irrigation was required in eight (33.3%) patients for the management of mild postoperative hematuria, with a median irrigation duration of 24 hours (IQR = 12-36 hours). All patients achieved clear urine before catheter removal.

Histopathological results

Pathological staging identified that 15 (62.5%) patients presented with T1 tumors, whereas nine (37.5%) patients had Ta lesions. Histopathological grading indicated low-grade tumors in 14 (58.3%) patients and high-grade tumors in 10 (41.7%) patients.

Adequate muscle sampling for staging was achieved in 22 (91.7%) patients, with two cases having insufficient detrusor muscle in the specimen for definitive T1 staging confirmation. All specimens provided adequate tissue for histological grading and staging assessment.

Comparative analysis

Comparison between single and multiple lesions revealed significant differences in analgesic requirements (Table 1). Single lesions required a median of 1.00 analgesic vials compared to 2.00 vials for multiple lesions (p = 0.017). This difference likely reflects the larger cumulative resection area and multiple surgical sites in patients with multiple tumors. Other parameters, including catheterization time, hospital stay, duration of analgesia, tumor size, and operative time, showed no statistically significant differences between single and multiple lesions, though multiple lesion cases showed trends toward higher resource utilization.

**Table 1:** Comparison of perioperative outcome variables between single and multiple bladder lesions. *: Multiple lesions group comprised four patients with two lesions, two patients with three lesions, and one patient with four lesions. Data are presented as median (IQR). Statistical significance is considered at p-values <0.05.

Variable	Single lesions (N = 17, 70.8%)	Multiple lesions (N = 7, 29.2%)	P-value (Mann-Whitney U test)
Catheterization time	2.00 (1.00–2.50)	1.00 (1.00–2.00)	0.144
Hospital stay	2.00 (1.00–3.00)	1.00 (1.00–1.00)	0.08
Analgesic vials used	1.00 (1.00–2.00)	2.00 (2.00–3.00)	0.017
Duration of analgesia	1.00 (1.00–1.00)	2.00 (1.00–2.00)	0.085
Tumor size	2.00 (1.50–2.00)	2.50 (2.00–3.00)	0.09
Operative time (minutes)	35.00 (27.50–37.50)	30.00 (25.00–40.00)	0.626

Analysis of outcome variables based on tumor staging (T1 vs. Ta) showed no statistically significant differences across all measured parameters. Similarly, comparison between low-grade and high-grade tumors demonstrated no statistically significant differences in any of the measured outcome variables. These findings suggest that the holmium laser technique provides consistent operative outcomes regardless of tumor biological characteristics.

Detailed tumor stage and grade comparison tables are provided in the Appendices, as they show no significant differences across measured parameters, representing important negative findings for clinical decision-making.

The comprehensive analysis demonstrates that holmium laser-assisted TURBT achieved successful tumor resection with acceptable complication rates (12.5% ONR, 8.3% bladder perforation) and favorable recovery parameters (median two-day catheterization, 1.5-day hospital stay) across different tumor characteristics and patient subgroups. The technique showed particular advantage for lateral wall tumors with reduced ONR rates compared to historical conventional TURBT data, while maintaining effective tissue sampling for accurate histopathological staging and grading.

## Discussion

The current investigation offers important data on clinical results and safety after holmium laser-assisted TURBT in people with superficial disease. These results add to the expanding literature that favors alternative energy methods over standard monopolar TURBT, especially in overcoming its known drawbacks. The male predominance observed in our study (62.5%) is consistent with established epidemiological patterns of bladder cancer and aligns with previous investigations in this field. Lu et al. [16] reported a higher male prevalence (76%), while Huang et al. [17] documented 68% of male patients. Similarly, Razzaghi et al. [18] found 89.7% male participants, and Zhou et al. [19] reported 66.7% males. This consistent male predominance across multiple studies reinforces the known gender predilection for bladder cancer, likely reflecting differences in occupational exposures, smoking patterns, and genetic susceptibility. The clinical presentation pattern in our study, with hematuria being the predominant symptom (58.3%), reflects the typical presentation of bladder cancer. The relatively high proportion of incidental findings (12.5%) may indicate improved screening practices and increased awareness of bladder cancer symptoms among both patients and healthcare providers.

The anatomical distribution of tumors in our study showed a predominance of right lateral wall involvement (37.5%), which differs from some previous reports. Lu et al. [16] found the trigone to be the most common location (93%), while our study showed a more diverse anatomical distribution. This variation may reflect differences in patient populations, referral patterns, or genetic factors influencing tumor development patterns. The prevalence of single lesions (70.8%) in our study is comparable to other investigations, suggesting that most patients present with localized disease amenable to complete resection. The median tumor size of 2.00 cm aligns with findings from Huang et al. [17], who reported a mean size of 1.53 ± 0.2 cm, indicating that most tumors in our series were within the optimal size range for laser resection techniques.

The median operative time of 32.50 minutes in our study was slightly longer than reported in previous investigations. Lu et al. [16] reported 23.4 ± 3.3 minutes, Huang et al. [17] documented 27.3 ± 6.62 minutes, and Zhou et al. [19] reported 28.8 ± 3.6 minutes. Several factors explain this difference: clinician proficiency, technical complexity, pathological characteristics, and organizational policies. The longer operative time in our series may also reflect the learning curve associated with implementing new surgical techniques and the meticulous approach required for en bloc resection with adequate margins.

The holmium laser’s unique properties, including its wavelength of 2,100 nm and strong water absorption characteristics, provide several advantages over conventional electrocautery. The limited tissue penetration depth of approximately 400 μm ensures precise tissue ablation while minimizing thermal damage to surrounding structures. The ability to generate steam bubbles that separate tissue layers while simultaneously achieving hemostasis contributes to the effectiveness of this technique.

A key benefit identified in our investigation was the accelerated healing time. The median bladder irrigation period of 2.00 days and inpatient duration of 1.50 days demonstrate superiority over earlier findings and standard TURBT outcomes. Huang et al. [17] reported longer catheterization (3.29 ± 0.46 days) and hospital stay (4.43 ± 0.55 days), while Lu et al. [16] documented even longer durations (3.6 ± 1.2 days and 7.9 ± 1.7 days, respectively). These improvements in recovery metrics likely reflect the superior hemostatic properties of the holmium laser and reduced tissue trauma compared to conventional techniques. The low complication rates observed in our study are particularly noteworthy. The bladder perforation rate of 8.3% is comparable to other laser series, with Huang et al. [17] reporting 7.1% and Razzaghi et al. [18] documenting 7.7%. The ONR rate of 12.5% compares favorably with conventional TURBT series, where rates can exceed 25% for lateral wall tumors. The absence of electrical current flow during laser procedures eliminates the risk of obturator nerve stimulation, making this technique particularly advantageous for lateral wall tumors.

The tumor staging distribution in our study, with 62.5% T1 and 37.5% Ta tumors, is consistent with typical superficial bladder cancer populations. The predominance of low-grade tumors (58.3%) suggests appropriate patient selection for this technique. The ability to obtain adequate tissue samples for histopathological evaluation is crucial for accurate assessment and prepping, and our study demonstrates the feasibility of achieving this goal with laser resection. The comparative analysis revealed that patients with multiple lesions required significantly more analgesic medication compared to those with single lesions (p = 0.017). This finding is clinically relevant as it suggests that tumor multiplicity may be associated with increased postoperative discomfort, possibly due to larger cumulative resection areas or the need for multiple resection sites. Interestingly, tumor staging and grading did not significantly influence operative outcomes or recovery parameters in our study. This suggests that the holmium laser technique provides consistent results across different tumor characteristics, supporting its broader applicability in superficial bladder cancer management. The holmium laser’s excellent hemostatic properties, demonstrated by minimal postoperative bleeding and reduced irrigation requirements, represent significant clinical advantages. The lower operating temperatures (40°C to 75°C) compared to conventional electrocautery (100°C to 300°C) result in reduced thermal injury and faster healing. The precise tissue ablation capabilities allow for accurate tumor margin control, which is crucial for oncological outcomes. However, certain limitations should be acknowledged. The technique requires specialized equipment and training, which may limit its widespread adoption. The learning curve associated with laser techniques may initially result in longer operative times. Additionally, the cost considerations of laser equipment and maintenance may influence institutional adoption decisions.

Our findings suggest that holmium laser-assisted TURBT represents a viable alternative to conventional techniques, particularly for patients with lateral wall tumors or those at high risk for conventional TURBT complications. The reduced complication rates, shorter recovery times, and favorable safety profile support its consideration as a first-line treatment option for appropriate patients. The growing body of evidence supporting laser techniques in bladder cancer management, as demonstrated by studies from Teng et al. [14], suggests that this technology may become increasingly important in urological practice. However, long-term oncological outcomes and recurrence rates require further investigation through larger prospective studies with extended follow-up periods.

This investigation has several important limitations. The modest sample size (n = 24) and single-center design limit generalizability across different practice settings and surgeon experience levels. The heterogeneous nature of the multiple lesions group (ranging from two to four tumors) precluded detailed subgroup analysis by tumor number. Our observational design prevents direct comparison with conventional TURBT in matched patient populations. Additionally, the learning curve effect, evidenced by decreased operative times with experience, may not be representative of widespread adoption scenarios. Short follow-up periods prevent assessment of long-term oncological outcomes and recurrence patterns, which remain crucial for technique validation.

## Conclusions

Holmium laser-assisted TURBT demonstrated acceptable operative outcomes with low complication rates, rapid recovery, and reasonable operative efficiency. The technique’s advantages, which include minimal tissue penetration, excellent hemostasis, and eliminated electrical current, address key limitations of conventional TURBT, particularly for lateral wall tumors. Key findings include reduced ONR rates in lateral wall resections and increased analgesic requirements for multiple lesions. While the technique requires specialized equipment, surgeon training, and involves higher initial costs, current evidence supports its consideration as a viable alternative for appropriately selected patients with superficial bladder carcinoma. Future research should focus on larger randomized controlled trials with extended follow-up, cost-effectiveness analyses, and standardized training protocols to facilitate broader adoption of this promising surgical technique.

## Appendices

**Table 2:** Comparison of perioperative outcome variables between T1 and Ta tumor staging using Mann-Whitney U test analysis. Data presented are as median (IQR). Statistical significance is considered at p-values <0.05.

Variable	T1 stage (N = 15, 62.5%)	Ta stage (N = 9, 37.5%)	P-value (Mann-Whitney U test)
Catheterization time	2.00 (1.00–3.00)	2.00 (1.00–2.00)	0.655
Hospital stay	2.00 (1.00–3.00)	1.00 (1.00–2.50)	0.35
Analgesic vials used	1.00 (1.00–2.00)	2.00 (1.00–2.50)	0.298
Duration of analgesia	1.00 (1.00–1.00)	1.00 (1.00–2.00)	0.276
Tumor size	2.00 (1.50–3.00)	2.00 (1.50–3.00)	0.726
Operative time (minutes)	35.00 (30.00–40.00)	30.00 (25.00–37.50)	0.562

**Table 3:** Comparison of perioperative outcome variables between low-grade and high-grade bladder tumors using Mann-Whitney U test analysis. Data presented are as median (IQR). Statistical significance is considered at p-values <0.05.

Variable	Low grade (N = 14, 58.3%)	High grade (N = 10, 41.7%)	P-value (Mann-Whitney U test)
Catheterization time	2.00 (1.00–2.00)	1.50 (1.00–3.00)	0.90
Hospital stay	2.00 (1.00–2.25)	1.00 (1.00–3.00)	0.68
Analgesic vials used	1.00 (1.00–2.00)	2.00 (1.00–3.00)	0.09
Duration of analgesia	1.00 (1.00–1.25)	1.00 (1.00–2.00)	0.417
Tumor size	2.00 (1.50–2.00)	2.50 (1.50–3.00)	0.153
Operative time (minutes)	30.00 (25.00–36.25)	35.00 (25.00–40.00)	0.719
